# Efficacy and safety of levornidazole disodium phosphate injection in patients with intra-abdominal infections caused by anaerobic bacteria: a multicenter, randomized, single-blind, phase IV, non-inferiority trial (ANAEROGUARD study)

**DOI:** 10.1186/s12879-026-12789-7

**Published:** 2026-02-28

**Authors:** Lin Chen, Lei Zhang, Rufu Chen, Zhiwei Zheng, Li Zhou, Xiufang Zhu, Yuanyuan Wang, Desheng Ni, Xiang Chen, Shuguang Zhao, Yuhuai Zhou, Chuxiao Shao, Jian Xu, Feng Sun, Guoying Wang, Tao Zhao, Xiaorong Li, Quan Wang, Shuanghai Liu, Jiaqing Cao, Yang Fu, Jianxin Ye, Bin Wang, Youming Ding, Wei Wang, Yunjie Zhang, Bo Huang, Jianzhao Huang, Wei Han, Wenxian Guan, Jialin Zhang, Zhongtao Zhang, Jiren Yu, Jiaojiao Yang, Yong Lan, Xiaoping Chen

**Affiliations:** 1 Department of Hepatobiliary Surgery, Hepatic Surgery Center, Tongji Hospital, Tongji Medical College, Huazhong University of Science and Technology, Wuhan, 430030 China; 2 Hubei Provincial Clinical Medical Research Center for Liver Surgery, Wuhan, 430030 China; 3 Hubei Key Laboratory of Hepato-Pancreato-Biliary Diseases, Wuhan, 430030 China; 4 Department of General Surgery, Shanxi Bethune Hospital, Taiyuan, 030032 China; 5 Department of Pancreatic Surgery, Guangdong Provincial People’s Hospital, Guangzhou, 510080 China; 6 Department of General Surgery, Rizhao People’s Hospital, Rizhao, 276800 China; 7 Department of Breast and Thyroid Vascular Surgery, Chizhou People’s Hospital, Chizhou, 247100 China; 8 Department of Gastroenterology, The First Hospital of Hebei Medical University, Shijiazhuang, 050031 China; 9 Department of Gastrointestinal Surgery, The First Hospital of Hebei Medical University, Shijiazhuang, 050031 China; 10 Department of Hepatobiliary, Pancreatic, Gastrointestinal Surgery, Jinhua People’s Hospital, Jinhua, 321000 China; 11 Department of General Surgery, Yixing People’s Hospital, Yixing, 214200 China; 12 Department of Emergency Medicine, Taihe County People’s Hospital, Fuyang, 236600 China; 13 Department of General Surgery, The First Affiliated Hospital of Shaoyang University, Shaoyang, 422000 China; 14 Department of General Surgery, Lishui People’s Hospital, Lishui, 323000 China; 15 Department of Hepatobiliary Surgery, Sichuan Provincial People’s Hospital, Chengdu, 610072 China; 16 Department of Gastrointestinal Surgery, The Second Affiliated Hospital of Kunming Medical University, Kunming, 650101 China; 17 Department of Hepatobiliary Surgery, The First Affiliated Hospital of Guangzhou Medical University, Guangzhou, 510120 China; 18 Department of Emergency Surgery, The First Affiliated Hospital of University of Science and Technology of China, Hefei, 230001 China; 19 Department of General Surgery, The Third Xiangya Hospital of Central South University, Changsha, 410013 China; 20 Department of Gastric and Colorectal Surgery, The First Hospital of Jilin University, Changchun, 130021 China; 21 Department of General Surgery, Jiangyin People’s Hospital, Jiangyin, 214400 China; 22 Department of Gastrointestinal Surgery, The Second Affiliated Hospital of Nanchang University, Nanchang, 330006 China; 23 Department of Gastrointestinal Surgery, The First Affiliated Hospital of Zhengzhou University, Zhengzhou, 450052 China; 24 Department of Gastrointestinal Surgery, The First Affiliated Hospital of Fujian Medical University, Fuzhou, 350005 China; 25 Department of Hepatobiliary Surgery, Dongyang People’s Hospital, Dongyang, 322100 China; 26 Department of Hepatobiliary Surgery, Renmin Hospital of Wuhan University, Wuhan, 430060 China; 27 Department of General Surgery, The Second Affiliated Hospital of Fujian Medical University, Quanzhou, 362000 China; 28 Department of Gastrointestinal and Hernia Surgery, Shandong University of Traditional Chinese Medicine, Jinan, 250355 China; 29 Department of General Surgery, Shanxi Provincial People’s Hospital, Taiyuan, 030012 China; 30 Department of Hepatobiliary Surgery, Guizhou Provincial People’s Hospital, Guiyang, 550002 China; 31 Department of General Surgery, Beijing Anzhen Hospital, Capital Medical University, Beijing, 100029 China; 32 Department of Gastrointestinal Surgery, Nanjing Drum Tower Hospital, The Affiliated Hospital of Nanjing University Medical School, Nanjing, 210008 China; 33 Department of Hepatobiliary Surgery, The First Hospital of China Medical University, Shenyang, 110001 China; 34 Department of General Surgery, Beijing Friendship Hospital, Capital Medical University, Beijing, 100050 China; 35 Department of General Surgery, The First Affiliated Hospital of Zhejiang University School of Medicine, Hangzhou, 310003 China; 36 Yangtze River Pharmaceutical Group Co,, Ltd., Taizhou, 225300 China; 37 Taizhou Pharmaceutical Association, Taizhou, 225300 China

**Keywords:** Nitroimidazole, Intra-abdominal infections, Bacteria, Anaerobic, Clinical trial, phase IV, Ornidazole

## Abstract

**Background and Purpose:**

Levornidazole is an ornidazole derivative effective against anaerobic pathogens. Levornidazole disodium phosphate, the disodium phosphate salt of levornidazole, exhibits higher water solubility compared to levornidazole. This study aimed to evaluate the efficacy and safety of levornidazole disodium phosphate in patients with intra-abdominal infections (IAIs) caused by anaerobic pathogens.

**Methods:**

This non-inferiority trial enrolled patients with IAIs in China, randomized 1:1 to either 1 g levornidazole disodium phosphate intravenously once daily (levornidazole group) or 0.5 g ornidazole and sodium chloride intravenously twice daily (control group) for 4–7 days. The primary endpoint was the clinical cure rate at the test-of-cure (TOC) visit. The non-inferiority margin was −8%. Secondary endpoints included the bacterial eradication rate, overall success rate at both TOC and the end of therapy (EOT) visits, and clinical cure rate at the EOT visit.

**Results:**

The full analysis set included 339 patients in the levornidazole group and 338 patients in the control group. At TOC, the clinical cure rates were 92.33% in the levornidazole group and 93.05% in the control group, with a between-group difference of −0.72% (95% CI: −4.85, 3.35). Clinical cure rates at EOT, bacteriological eradication rates, and overall success rates at EOT and TOC were similar in the two groups. The incidence of adverse drug reactions was 9.86% (34/345) in the levornidazole group and 12.17% (42/345) in the control group.

**Conclusions:**

Once-daily levornidazole disodium phosphate was non-inferior to twice-daily ornidazole and sodium chloride for treating IAI, offering a more convenient, better-tolerated option due to its lower dosing frequency.

**Supplementary Information:**

The online version contains supplementary material available at 10.1186/s12879-026-12789-7.

## Introduction

Intra-abdominal infection (IAI) is a common surgical emergency and a leading cause of non-traumatic in-hospital mortality worldwide [1]. IAI is classified as either primary or secondary peritonitis, with the secondary type being more prevalent. Secondary peritonitis primarily arises from gastrointestinal perforations (e.g., peptic ulcer, appendicitis, diverticulitis), complications of bowel obstruction, biliary tract infections, hepatic abscesses, and iatrogenic causes [2]. Anaerobic bacteria are typically present on mucosal surfaces. These bacteria can translocate and cause infection when the protective barrier is compromised, or when local tissue hypoxia and barrier disruption occur due to surgical trauma [3, 4]. The gut and genitourinary tract host hundreds of bacterial species, and IAIs often result from mixed infections involving both anaerobic and aerobic organisms. This complexity necessitates antimicrobial regimens that provide broad-spectrum coverage while considering safety [5–7]. The management of IAIs typically involves antimicrobial treatments effective against common pathogens, including typical Gram-negative Enterobacteriaceae, Gram-positive cocci, and obligate anaerobes [8]. Among these agents, nitroimidazoles (metronidazole, ornidazole, or levornidazole) have narrow-spectrum activity against anaerobes and can offer targeted therapeutic options for anaerobic infections following abdominal surgery [8–10].

Levornidazole, the S-enantiomer of ornidazole, is a third-generation nitroimidazole derivative that is commonly used in clinical practice in China. It is known for its strong efficacy against anaerobic bacteria and its relatively low neurotoxicity profile [11–14]. The antimicrobial action of levornidazole is based on the reduction of its nitro group under anaerobic conditions, leading to the formation of active metabolites that disrupt bacterial DNA structure [15]. However, levornidazole is poorly soluble in water and requires an acidic vehicle with a pH of 2–4 for solubilization and stability. This requirement can result in infusion-site pain and phlebitis [16]. Consequently, there is an urgent need for anti-anaerobic agents that are safe and easy to administer, particularly in the critical context of peritonitis.

Levornidazole disodium phosphate (XINRUI^®^) is a phosphate ester derivative of the levo-isomer of ornidazole, developed through structural modifications to achieve multiple pharmacological improvements. The disodium phosphate salt is used not to change the intrinsic antimicrobial activity, but to optimize solubility, formulation safety, and IV administration characteristics, acting as a rapidly cleaved phosphate prodrug of levornidazole [17]. Pharmacokinetic work in animals shows that levornidazole disodium phosphate is rapidly metabolized to levornidazole after administration, with exposure increasing proportionally with dose [17]. In 2021, levornidazole disodium phosphate received approval from the National Medical Products Administration (NMPA) for the treatment of various anaerobic bacterial infections, amoebiasis, and for the prevention and management of anaerobic infections following surgical procedures [18]. The levornidazole disodium phosphate formulation has a pH range of 5.0 to 5.4, which is closer to physiological conditions, potentially reducing the incidence of infusion-site pain and phlebitis [19, 20]. In a randomized phase II trial (unpublished data; http://www.chictr.org.cn ChiCTR2300073123; available as a preprint [21]) involving 146 patients with pelvic anaerobic infections, the clinical cure rate was 83.76% for the once-daily 1 g levornidazole disodium phosphate group (*n* = 50), which was numerically higher than the cure rate of 82.61% observed in the twice-daily 0.5 g group (*n* = 47), but the numerically lowest clinical cure rate was observed in the levornidazole and sodium chloride group (control group, 77.55%, *n* = 49). The incidence of adverse drug reactions (ADRs) was comparable among the three groups: 38.78% (control group), 18.37% (1 g/Qd levornidazole disodium phosphate group), and 21.74% (0.5 g/12 h levornidazole disodium phosphate group). However, data on the efficacy and safety of levornidazole disodium phosphate in patients with IAIs remains limited.

This phase IV study aimed to evaluate the efficacy and safety of levornidazole disodium phosphate in patients with IAIs caused by anaerobic pathogens. The results could provide valuable insights into the use of levornidazole disodium phosphate in the clinical setting.

## Methods

### Study design and participants

This phase IV clinical trial was designed as a prospective, multicenter, randomized, single-blind, active-controlled, parallel-group, non-inferiority study conducted at 32 research centers across China from May 10, 2023, to October 14, 2024. The study was conducted in accordance with the Declaration of Helsinki and the International Council for Harmonisation’s Good Clinical Practice guidelines. Approval was obtained from the Ethics Committee of Clinical Trials, Huazhong University of Science and Technology ([2022] Ethics Review No. (176)-1) and each participating center. All patients provided written informed consent. The trial was prospectively registered with the Center for Drug Evaluation (CDE) of the National Medical Products Administration (NMPA) (CTR20223205; December 23, 2022) and retrospectively registered at ClinicalTrials.gov (NCT06828874; February 17, 2025).

The Supplementary Materials present the complete list of selection criteria. The key inclusion criteria required participants to be adults aged 18 to 75 years, regardless of sex. Patients were eligible if they were scheduled to undergo minimally invasive surgery (laparoscopic or robot-assisted), open surgery, or percutaneous drainage for IAIs deemed by the investigator to be caused by anaerobic bacteria [22]. Meanwhile, patients had to meet at least one of the following criteria: 1) at least two of the four preoperative indicators within 24 hours: i) abdominal pain or abdominal tenderness with fever (axillary temperature ≥37.5°C), ii) white blood cell count ≥10 × 10^9^/L, iii) elevated C-reactive protein above the upper limit of normal, or iv) elevated procalcitonin above the upper limit of normal, 2) radiological evidence of IAI confirmed by computed tomography or ultrasound. Additionally, patients were required to start or plan to start the investigational drug within 24 hours postoperatively. They also needed to provide voluntary, written informed consent.

The key exclusion criteria were 1) known or suspected hypersensitivity to nitroimidazole-class agents, 2) participation in another clinical study involving investigational drugs within 3 months before screening, 3) concurrent use of other medications or presence of comorbidities that may interfere with the assessment of the investigational drug’s safety or efficacy, or the presence of a high risk of significant drug-drug interactions due to concomitant treatments, 4) concomitant infections at sites outside the abdominal cavity, 5) conditions deemed by the investigator to require alternative broad-spectrum antibiotics with anti-anaerobic activity due to disease severity, 6) receipt of antibiotic therapy within 48 hours before randomization, or 7) any other condition that, in the judgment of the investigator, rendered the patient unsuitable for participation in the clinical trial.

### Procedure

Randomization was conducted by the investigator using a central randomization system (IWRS). A block-randomized method (block size 8) was used, with eligible participants randomly assigned in a 1:1 ratio to receive either levornidazole disodium phosphate (levornidazole group) or ornidazole and sodium chloride (control group).

Participants in the levornidazole group received intravenous infusions of levornidazole disodium phosphate (XINRUI^®^; 0.125 g/vial, Yangtze River Pharmaceutical Group Jiangsu Zilong Pharmaceutical Co., Ltd.) starting on the first post-surgery day. They were administered once daily at a dose of 1 g per infusion, with each infusion lasting at least 60 minutes. Participants in the control group received intravenous infusions of ornidazole and sodium chloride injection (100 mL: 0.5 g ornidazole, Sichuan Kelun Pharmaceutical Co., Ltd.), starting on the first day post-surgery, and were administered twice daily at a dose of 0.5 g per infusion. After 4 days of treatment, the investigator determined whether to continue the study drug based on the patient’s clinical response, with treatment extending up to day 7. In addition to the study drug, supportive therapy and concomitant medications were at the investigator’s discretion. The investigator selected drugs targeting aerobic bacteria or fungi based on bacteriological results, susceptibility testing, and guidelines [22]. During the study, the use of other antimicrobial agents targeting anaerobes or broad-spectrum antibiotics with anti-anaerobic activity was prohibited. Cases would be classified as “clinical failure” and included in the full analysis set (FAS) if the patient’s IAI persisted (including persistence of abdominal pain or with axillary temperature ≥37.5 °C, or white blood cell count≥10 × 10^9^/L) or has worsened and would require antibiotic therapy beyond the planned 7-day course, indicating the development of an infectious complication. The outcome evaluators who evaluated the subjects’ clinical symptoms and laboratory examinations, including the presence or resolution of patient-reported abdominal pain, alleviation or disappearance of tenderness on abdominal physical examination, remission of fever symptoms, and normalization of body temperature and total white blood cell count, at the end of therapy (EOT) and test of cure (TOC) visits, remained blinded throughout the study.

Participants were required to complete visits on postoperative Day 1, postoperative Day 3, and at the EOT during treatment, as well as a TOC visit within 5 to 10 days after EOT.

### Assessment and endpoint

The primary endpoint was the clinical cure rate at the TOC visit. The secondary endpoints included the clinical cure rate, bacterial eradication rate, and overall success rate at the EOT visit, and the bacterial eradication rate and overall success rate at the TOC visit.

Clinical cure was defined as the complete resolution or return to normal of all baseline symptoms and signs related to the infection, along with normalization of non-microbiological laboratory indicators 1) resolution or improvement of abdominal pain, 2) axillary temperature ≤37.5 °C, and 3) white blood cell count < 10 × 10^9^/L. Additionally, if postoperative imaging (CT or ultrasound) was performed at the efficacy evaluation time point, the results had to show no evidence of active infection or return to normal.

Clinical failure was defined as the persistence or incomplete resolution of all symptoms and signs at EOT or TOC visits (failure to meet the above criteria for clinical cure). It can also include any worsening of clinical manifestations, the appearance of new symptoms or signs related to IAIs, and/or the necessity to administer additional antibacterial agents targeting anaerobic bacteria. Additionally, patients who showed partial improvement but still required modifications or an increase in their anaerobic treatment regimen should also be classified as experiencing clinical failure. The clinical cure rate was calculated as the percentage of participants with a clinical cure among all participants.

Bacteriological efficacy was assessed only in cases with positive anaerobic cultures from intraoperative specimens at baseline, and the following five determinations were made regarding clinical cure and microbiological findings. Eradication indicated the absence of the original pathogen in post-treatment cultures from the primary infection site. For patients achieving clinical cure, bacteriological results were considered presumed eradication if the resolution of signs and symptoms renders culturable material unobtainable (e.g., sputum, skin pus, or secretions) or if specimen collection methods were deemed excessively invasive for recovered patients. Non-eradication indicated continued isolation of the original pathogen from the primary infection site. For patients deemed clinical failures, bacteriological outcome was designated as presumed non-eradication if cultures were not performed or could not be obtained. Other categories encompassed microbiological outcomes such as microbial turnover, superinfection, relapse, or colonization. The eradication rate was calculated as the percentage of participants with eradication or presumed eradication among those with baseline-positive cultures. Composite efficacy was assessed only in cases with positive anaerobic cultures from intraoperatively obtained baseline specimens and was determined by an integrated analysis of changes in participants’ symptoms, signs, imaging, laboratory tests, and pathogen assessments before and after treatment. The following classification was used. 1) Cure: patients with clinical resolution at EOT or TOC, and bacteria were eradicated or presumed eradicated. 2) Failure: patients were classified as clinical failure at EOT or TOC, bacteria were not eradicated, presumed non-eradicated, or both. The overall success rate was calculated as the percentage of participants with a clinical cure among those with baseline-positive cultures.

Safety assessments included physical examinations, vital signs, electrocardiograms, laboratory tests, and other adverse events, serious adverse events, and ADR throughout the study. A treatment-emergent adverse event (TEAE) was defined as an adverse event that occurred during the use of the study drug. A serious adverse event (SAE) was defined as any untoward medical occurrence that at any dose: (1) results in death; (2) is life-threatening; (3) requires inpatient hospitalization or prolongation of existing hospitalization; (4) results in persistent or significant disability/incapacity; (5) is a congenital anomaly/birth defect. ADR was defined as any adverse event that in the investigator’s opinion may have been caused by the study drugs with reasonable possibility. Adverse events were graded according to the NCI-CTC AE V5.0.

### Statistical analysis

The randomized set (RAND) included all participants randomized into the study. The FAS included all randomized participants who received at least 1 dose of the study drug and had post-treatment efficacy data, in accordance with the intention-to-treat (ITT) principle. The per-protocol set (PPS) was a subset of the FAS, consisting of participants who adhered to the protocol, completed the primary endpoint evaluation, and did not deviate significantly from the study protocol. The FAS and PPS were used for efficacy analysis. The safety set (SS) included all participants randomized into the study who received at least one dose of the study drug and had post-treatment safety data. The SS were used for safety assessments.

Based on previous studies [23, 24], assuming a clinical cure rate of 85% for both the levornidazole and control groups, a non-inferiority margin of −8% [25], a one-sided alpha level of 0.025, a power of 80%, and a 1:1 sample allocation ratio, the required sample size per group was 313 participants. With a 10% dropout rate, approximately 348 participants per group were needed, yielding a total sample size of 696.

The primary endpoint was analyzed in the FAS and PPS. In the case of concomitant events, defined as the use of protocol-prohibited medications or treatments between the first administration of the study drug and the clinical efficacy evaluation within 5–10 days after EOT, a composite variable strategy was employed to classify the clinical efficacy results as failure. Prohibited medications refer to other anti-anaerobic agents, and broad-spectrum antibiotics with anti-anaerobic activity. The latter excludes the aerobic-coverage antibiotics specified in the trial protocol, namely first-, second-, and third-generation cephalosporins, aztreonam, quinolones, and aminoglycosides. Missing data following the handling of concomitant events were imputed using the multiple imputation (MI) method based on the missing at random (MAR) mechanism, with 10 imputations for the clinical efficacy assessment at TOC. The variables used in the imputation model were the treatment group and the clinical cure assessment at EOT. For each imputed dataset, the Newcombe-Wilson method was used to calculate the 95% confidence interval (CI) for the between-group rate difference, and the results from all imputed datasets were combined to obtain the overall rate difference and 95% CI. If the lower limit of the 95% CI was greater than −8%, the levornidazole group was noninferior to the control group. The sensitivity analysis for the primary endpoint was based on the data after handling concomitant events (without imputation) and used the Newcombe-Wilson method to calculate the 95% CI for the between-group rate difference. The secondary endpoints were analyzed in the FAS and PPS. Missing data were not imputed. The χ^2^ test was used to compare between-group differences at the corresponding visit points, and the Newcombe-Wilson method was employed to calculate the 95% CI for the between-group rate difference. Subgroup analyses of the primary endpoint were performed according to age (≤65 or > 65 years), etiology (appendicitis, gastrointestinal perforation, biliary tract infection, or liver abscess), surgical method (minimally invasive surgery, open surgery, or percutaneous drainage), and the type of aerobic bacterial antibiotics used in combination (quinolones, aminoglycosides, β-lactams, or others). In the post hoc analyses, the primary endpoint was evaluated according to the following subgroups: age > 60 years; suppurative appendicitis; gangrenous perforated appendicitis; peri-appendicular abscess; age ≥65 years with appendicitis as the etiology; age > 45 years with appendicitis or gastrointestinal perforation as the etiology, and no use of β-lactams or other aerobic bacterial antibiotics in combination.

Statistical analyses were conducted using SAS 9.4 or higher (SAS Institute Inc., Cary, NC, USA). Two-sided tests were used, with P-values < 0.05 considered statistically significant.

### Reporting

This study was reported in accordance with the Consolidated Standards of Reporting Trials (CONSORT) guideline. The completed CONSORT checklist has been submitted as a supplementary file.

## Results

### Characteristics of the participants

**Fig. 1:**
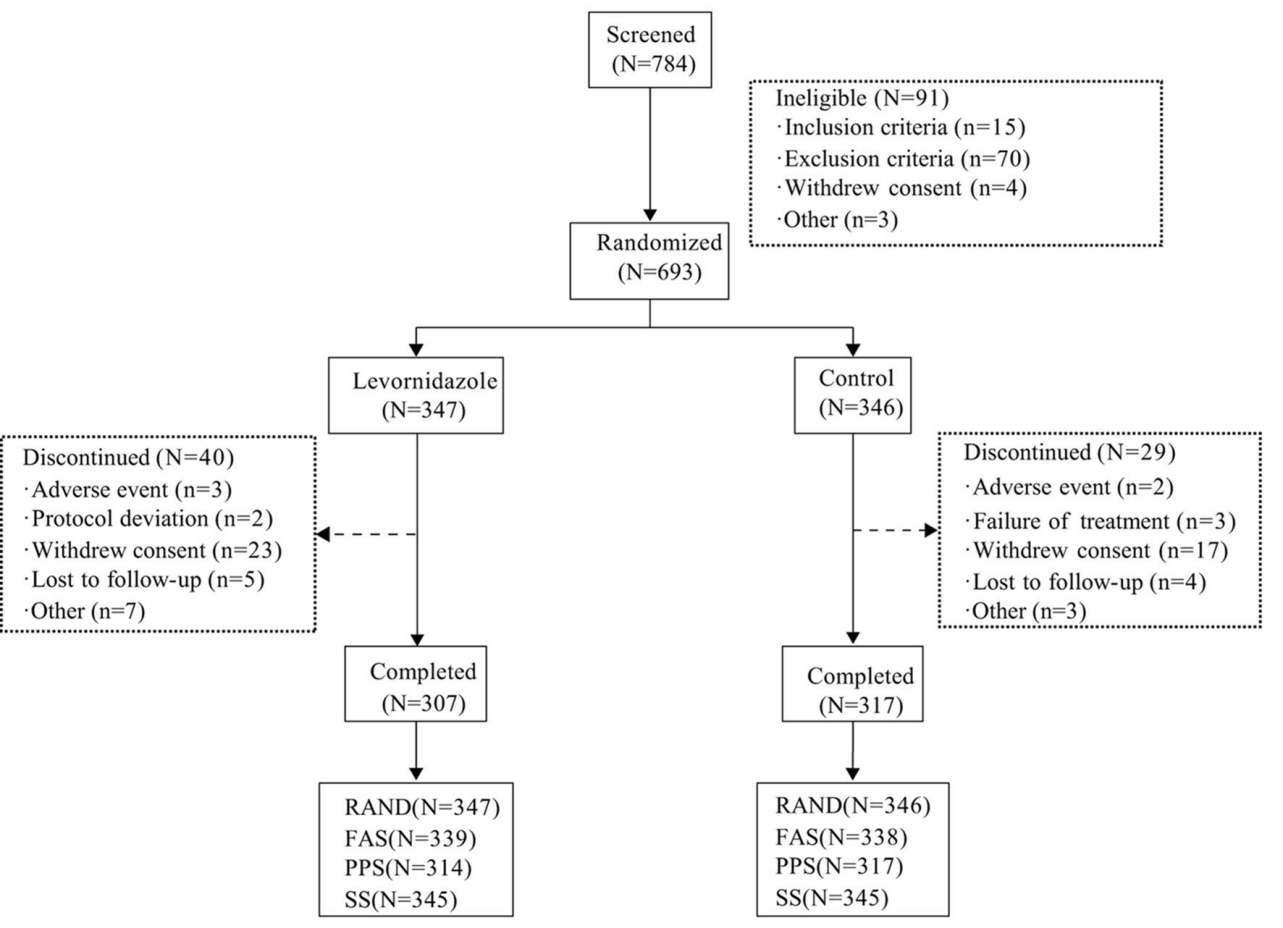
Patient flowchart

Following screening, 693 patients were randomized (RAND set): 347 to levornidazole and 346 to the control. The FAS comprised 677 participants (levornidazole: 339; control: 338), while PPS included 631 participants (levornidazole: 314; control: 317). The SS contained 690 participants, with 345 in each group. The screening process is shown in Fig. 1.

The FAS demonstrated balanced demographic and clinical profiles. The mean age of the participants in the levornidazole and control groups was 42.35 ± 15.21 and 42.91 ± 14.91 years, respectively, with similar male representation (56.93% vs. 55.62%). Surgical etiologies showed balanced distribution: appendicitis (81.12% vs. 83.14%), gastrointestinal perforation (4.13% vs. 2.66%), and biliary tract infection/liver abscess (14.75% vs. 14.20%). The proportions of participants undergoing minimally invasive surgery (97.35% vs. 97.04%), open surgery (1.18% vs. 1.78%), and percutaneous drainage (1.47% vs. 1.18%) were also similar between groups. β-lactams/other antibiotics constituted the primary concomitant aerobic antibacterials (levornidazole: 88.03%; control: 91.14%; Table 1). Bacteriological and susceptibility results were presented in Table S1.

**Table 1:** Baseline demographics and characteristics in FAS

Characteristics	Levornidazole group (n = 339)	Control group (n = 338)	P
Age, years	42.35±15.21	42.91±14.91	0.6286
Age, n (%)			0.8023
≤65	306 (90.27)	307 (90.83)	
>65	33 (9.73)	31 (9.17)	
Sex, n (%)			0.7310
Male	193 (56.93)	188(55.62)	
Female	146 (43.07)	150(44.38)	
BMI, kg/m2	23.77±3.63	23.95±3.49	0.5457
Etiology, n (%)			0.5513
Appendicitis	275 (81.12)	281 (83.14)	
Gastrointestinal perforation	14 (4.13)	9 (2.66)	
Biliary tract infection or liver abscess	50 (14.75)	48 (14.20)	
Surgical method, n (%)			0.8206
Minimally invasive surgery	330 (97.35)	328 (97.04)	
Open surgery	4 (1.18)	6 (1.78)	
Percutaneous drainage	5 (1.47)	4 (1.18)	
Concomitant aerobic antibiotic class, n (%)			0.2002
Quinolones	32 (13.68)	23 (9.70)	
Aminoglycosides	1 (0.43)	0 (0)	
β-lactams or others	206 (88.03)	216 (91.14)	

BMI: body mass index

### Efficacy

**Fig. 2:**
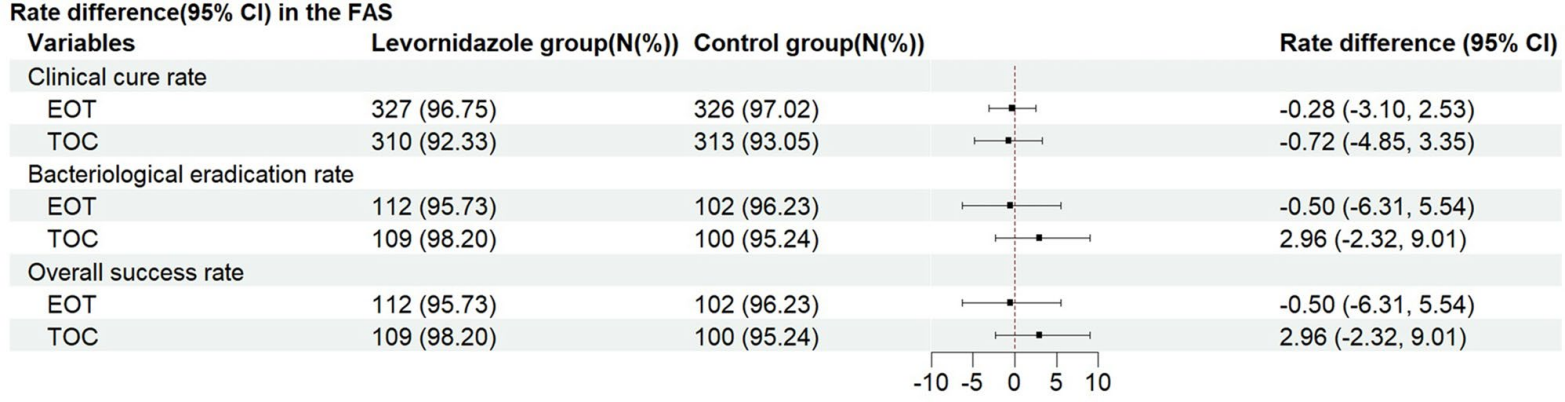
Forest plot of efficacy metrics

At the TOC visit in the FAS, the clinical cure rate in the levornidazole group was 92.33%, and in the control group, it was 93.05%. The between-group rate difference was −0.72% (95% CI: −4.85, 3.35), with the lower limit of the 95% CI greater than −8%, establishing non-inferiority (Table 2 and Fig. 2). In the PPS population, the clinical cure rate was 97.77% (307/314) in the levornidazole group and 97.16% (308/317) in the control group. The rate difference was −0.61% (95% CI: −2.05, 3.33; Table S2). In the FAS, the sensitivity analysis of the primary endpoint showed a clinical cure rate of 92.81% (310/334) in the levornidazole group and 93.15% (313/336) in the control group, with a between-group rate difference of −0.34% (95% CI: −4.32, 3.62; Table S3).

**Table 2:** Efficacy metrics in the FAS

Variables	Visits	Levornidazole group	Control group	Rate difference (95% CI)
Clinical cure rate, n (%)	TOC^*^	310 (92.33) n = 334	313 (93.05) n = 336	−0.72 (−4.85, 3.35)
	EOT	327 (96.75) n = 338	326 (97.02) n = 336	−0.28 (−3.10, 2.53)
Bacteriological eradication rate, n (%)	TOC	109 (98.20) n = 111	100 (95.24) n = 105	2.96 (−2.32, 9.01)
	EOT	112 (95.73) n = 117	102 (96.23) n = 106	−0.50 (−6.31, 5.54)
Overall success rate, n (%)	TOC	109 (98.20) n = 111	100 (95.24) n = 105	2.96 (−2.32, 9.01)
	EOT	112 (95.73) n = 117	102 (96.23) n = 106	−0.50 (−6.31, 5.54)

*Missing data (5 in levornidazole, 2 in control groups) were imputed using multiple imputation (MI). CI: confidence interval; EOT: end of therapy; TOC: test of cure

At the EOT visit in the FAS, 327 participants (96.75%) in the levornidazole group and 326 (97.02%) in the control group achieved clinical cure, with a rate difference of −0.28% (95% CI: −3.10, 2.53). The bacteriological eradication rate in the levornidazole group was 95.73% (112/117), and in the control group, 96.23% (102/106), with a difference of −0.50% (95% CI: −6.31, 5.54). A detailed breakdown of bacteriological eradication outcomes, specifying presumed eradication versus actual eradication, is shown in Supplementary Table S4. The overall efficacy evaluation showed that the overall success rate in the levornidazole group was 95.73% (112/117) and in the control group, it was 96.23% (102/106), with a rate difference of −0.50% (95% CI: −6.31, 5.54; Table 2 and Fig. 2).

At the TOC visit, the bacteriological eradication rate was 98.20% (109/111) in the levornidazole group and 95.24% (100/105) in the control group, with a between-group rate difference of 2.96% (95% CI: −2.32, 9.01). The overall efficacy evaluation at TOC showed a success rate of 98.20% (109/111) in the levornidazole group and 95.24% (100/105) in the control group, with a difference of 2.96% (95% CI: −2.32, 9.01; Table 2 and Fig. 2).

In the PPS population, the results of the secondary endpoints at different visit points were consistent with those in the FAS (Table S2).

**Fig. 3:**
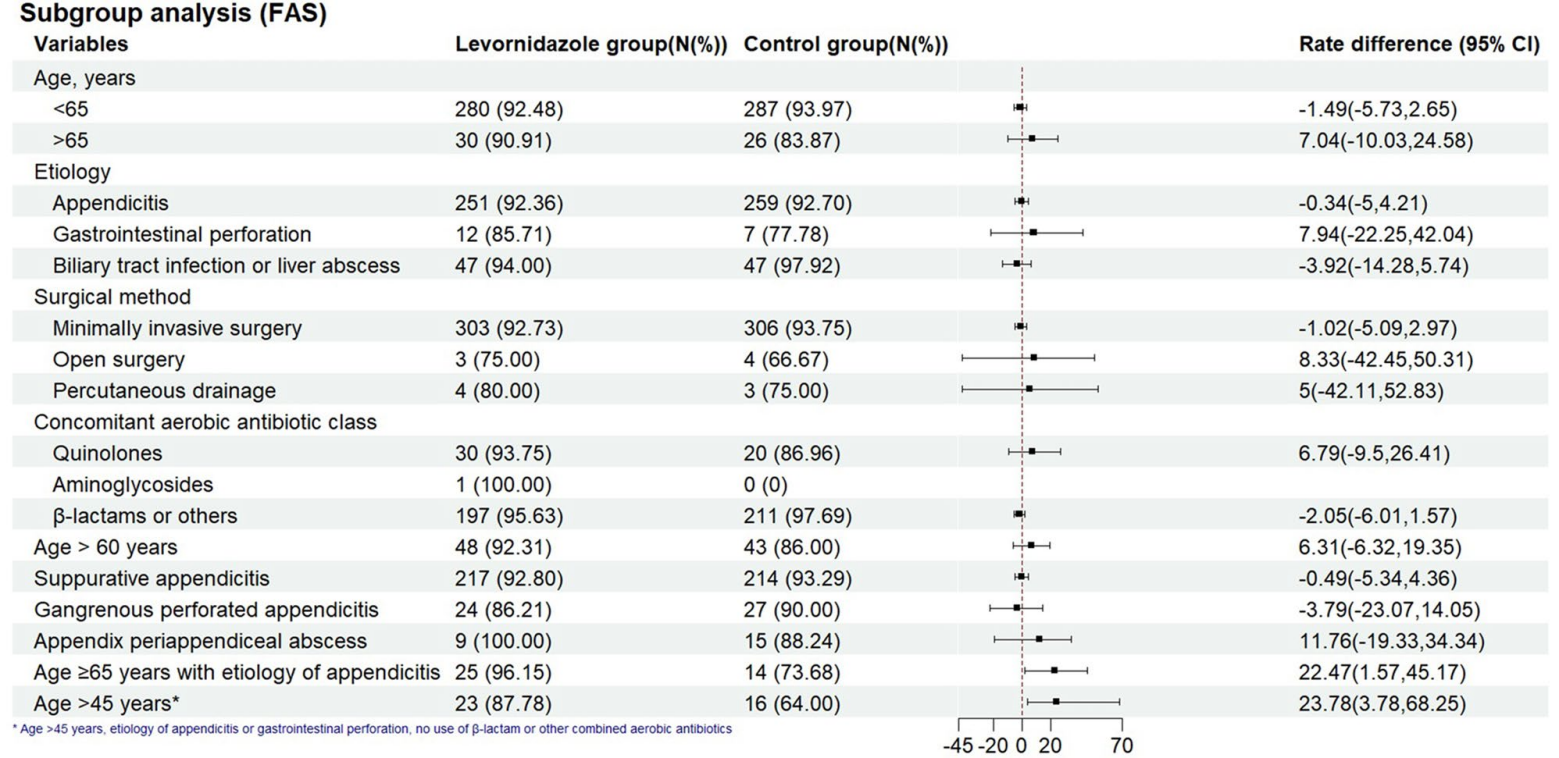
Forest plot of subgroup analysis

In subgroups with age ≤65 years, appendicitis as the cause, minimally invasive surgery, and β-lactam or other aerobic bacterial antibiotics, the between-group differences in clinical cure rate at the TOC visit were not statistically significant (Fig. 3 and Table S5). Post hoc analysis showed that in the subgroup of patients aged ≥65 years with appendicitis and in the subgroup of patients aged > 45 years with appendicitis or gastrointestinal perforation who did not use β-lactam or other types of aerobic bacterial antibiotics, the clinical cure rate at the TOC visit was higher in the levornidazole group than in the control group (Table S6 and Figure S1).

### Safety

In the levornidazole group, the median exposure was 4.0 g (Q1, Q3: 4, 5) with a median exposure duration of 4 days (Q1, Q3: 4, 5). In the control group, the median exposure was 4.0 g (Q1, Q3: 4, 4) with a median exposure duration of 5 days (Q1, Q3: 4, 5). During treatment, the incidence of TEAEs was 46.67% (161/345) in the levornidazole group and 45.80% (158/345) in the control group; the incidence of SAEs was 1.45% (5/345) in the levornidazole group and 0.58% (2/345) in the control group. In the levornidazole group, the incidence of TEAEs leading to drug interruption, drug discontinuation, trial withdrawal, and grade ≥3 TEAEs were 0.29% (1/345), 2.32% (8/345), 2.03% (7/345), and 1.74% (6/345), respectively. In the control group, these rates were 0.87% (3/345), 1.74% (6/345), 0.58% (2/345), and 1.16% (4/345), respectively.

The incidence of ADRs in the levornidazole group was 9.86% (34/345), and in the control group, 12.17% (42/345). The incidence of ADRs of grade ≥3 was 0.29% (1/345) in the levornidazole group and 0% in the control group. No drug-related SAEs, TEAEs leading to death, or fatal ADRs were reported in either treatment group (Table 3).

**Table 3:** Adverse events and adverse events of particular interest in the SS set

Items	Levornidazole group (n = 345)	Control group (n = 345)
TEAEs, n (%)	161 (46.67)	158 (45.80)
SAEs, n (%)	5 (1.45)	2 (0.58)
ADRs, n (%)	34 (9.86)	42 (12.17)
Grade ≥3 TEAEs, n (%)	6 (1.74)	4 (1.16)
Grade ≥3 ADRs, n (%)	1 (0.29)	0 (0.00)
SAE related to the study drug, n (%)	0 (0.00)	0 (0.00)
TEAE leading to drug interruption, n (%)	1 (0.29)	3 (0.87)
ADR leading to drug interruption, n (%)	1 (0.29)	2 (0.58)
TEAE leading to drug discontinuation, n (%)	8 (2.32)	6 (1.74)
ADR leading to drug discontinuation, n (%)	5 (1.45)	2 (0.58)
TEAE leading to trial withdrawal, n (%)	7 (2.03)	2 (0.58)
ADR leading to trial withdrawal, n (%)	5 (1.45)	1 (0.29)
TEAE leading to death, n (%)	0 (0.00)	0 (0.00)
ADR leading to death, n (%)	0 (0.00)	0 (0.00)
Phlebitis on day 1 postoperatively, n (%)	1 (0.29)	0 (0.00)
Phlebitis on day 3 postoperatively, n (%)	2 (0.59) [*n* = 340]	1 (0.30) [*n* = 338]
Phlebitis at the EOT visit, n (%)	0 (0.00) [*n* = 333]	1 (0.30) [*n* = 332]

TEAE: treatment-emergent adverse event; SAE: serious adverse event; ADR: adverse drug reaction; EOT: end of therapy

The most common ADRs during treatment in levornidazole group and control group were liver dysfunction (1.16% vs. 2.03%), nausea (1.16% vs. 1.45%), diarrhea (0.58% vs. 1.45%), elevated alanine aminotransferase (0.58% vs. 1.16%), elevated aspartate aminotransferase (0.58% vs. 1.16%), fever (0.29% vs. 0.87%), limb pain (0.87% vs. 0.87%), dizziness (0 vs. 0.87%), and vomiting (0.87% vs. 0.58%) (Table 4).

**Table 4:** Common ADR in the SS set

Items	Levornidazole group (n = 345)	Control group (n = 345)
Nausea, n (%)	4 (1.16)	5 (1.45)
Diarrhea, n (%)	2 (0.58)	5 (1.45)
Vomiting, n (%)	3 (0.87)	2 (0.58)
Alanine aminotransferase elevation, n (%)	2 (0.58)	4 (1.16)
Aspartate aminotransferase elevation, n (%)	2 (0.58)	4 (1.16)
Liver dysfunction, n (%)	4 (1.16)	7 (2.03)
Fever, n (%)	1 (0.29)	3 (0.87)
Limb pain, n (%)	3 (0.87)	3 (0.87)
Dizziness, n (%)	0	3 (0.87)

On postoperative day 1, day 3, and at EOT, the incidence of phlebitis was 0.29% (1/345), 0.59% (2/340), and 0 in the levornidazole group, respectively; and 0, 0.30% (1/345), and 0.30% (1/338) in the control group, respectively, with no statistically significant between-group differences (*p* > 0.05). During treatment, the incidence of adverse events related to liver function, inflammatory markers, and the nervous system was 2.32% (8/345) in the levornidazole group and 5.22% (18/345) in the control group, with a statistically significant difference between groups (difference of −2.90%, 95% CI: −5.73 to −0.07, *p* = 0.0456).

## Discussion

This non-inferiority randomized controlled trial demonstrated that levornidazole disodium phosphate was non-inferior to ornidazole and sodium chloride in clinical cure rate within 5 to 10 days after EOT in patients with IAI. Both treatment groups showed comparable clinical cure rates, bacterial eradication rates, and overall success rates at the EOT and TOC visits. In addition, TEAE rates were comparable across groups, with both groups exhibiting low SAE rates. These results suggest that levornidazole disodium phosphate is a safe and effective option for treating IAI.

A previous single-arm trial investigating the pharmacokinetic profile of levornidazole and sodium chloride included IAI patients with a mean age of 39.14 ± 13.05 years. The underlying causes of infection were appendicitis (85.7%) and gastrointestinal perforation (14.3%), which are consistent with the population in the current study. This multiple-dose trial suggested that levornidazole 0.5 g twice daily for 3 to 7 days was an effective treatment for abdominal anaerobic infection, achieving a clinical cure rate of 100% [26]. Another randomized controlled trial conducted by Ma et al. also demonstrated that levornidazole (*n* = 70) and ornidazole (*n* = 73), both administered at a dose of 0.5 g twice daily for 3 to 7 days, achieved clinical efficacy rates of 80 and 81%, and bacterial clearance rates of 97 and 92%, respectively, in patients with pelvic anaerobic infections [12]. This study showed similar clinical cure rates and bacteriological eradication rates with levornidazole disodium phosphate in patients with anaerobic infections. Moreover, the bacterial eradication rate increased from EOT to TOC visit in the levornidazole group, whereas a modest decline was observed in the control group during the same interval. The present study also showed that levornidazole disodium phosphate was noninferior to ornidazole across key demographic and clinical subgroups, including age, surgical etiologies, surgical method, and the use of other antibiotics against aerobic bacteria. Post hoc analyses suggested numerically higher clinical cure rates in patients aged > 45 and ≥65 years. It could be attributed to age-related differences in the pathogen profile of intra-abdominal infections, changes in the host immune response, or potentially more nuanced pharmacokinetic properties in older patients, all of which warrant further investigation. Nonetheless, these results should be interpreted with caution. The multicenter, randomized controlled design of this study enhanced the generalizability of our findings. Additionally, the high completion rate and low loss to follow-up minimized potential attrition bias, thereby strengthening the robustness of the outcomes. These results could provide valuable guidance for clinical practice.

In the present study, the overall incidence of TEAEs was similar between the two groups. Still, the ADR rate was lower in the levornidazole group compared with the control group. Both groups had a low incidence of phlebitis, with phlebitis occurring earlier in the levornidazole group and later in the control group, including after treatment completion. The complexity of concomitant medications may explain the lack of differences in phlebitis and overall AEs between the two groups. Indeed, Ma et al. [12] reported markedly lower AE rates with levornidazole and sodium chloride (3%) than with ornidazole (22%). When considering other third-generation nitroimidazoles in the treatment of IAI (e.g., ornidazole and tinidazole), the rates of gastrointestinal AEs are 5%-15% and central nervous system AEs are 1%-10%, while neurotoxicity and hepatotoxicity are rare ( < 0.1%) [27–29]. Similarly, this study demonstrated that levornidazole disodium phosphate has a distinct safety advantage in organ-specific toxicity profiles compared to ornidazole. Importantly, adverse events affecting hepatic function, inflammatory markers, and the neurologic system were significantly lower with levornidazole disodium phosphate than with ornidazole. In summary, nitroimidazole antibiotics have a favorable safety profile for the management of pelvic or intra-abdominal anaerobic bacterial infections. Still, head-to-head comparisons between levornidazole disodium phosphate and other nitroimidazoles may be warranted to more definitively establish comparative safety.

This study had limitations. The study primarily involved a Chinese population, and its findings may not be generalizable. IAI frequently involves mixed infections, necessitating empirical anaerobic therapy plus tailored antimicrobial coverage for aerobic/facultative anaerobic bacteria or fungi based on clinical judgment and susceptibility data; this therapeutic heterogeneity may confound safety outcomes due to challenges in definitive adverse event attribution, though subgroup analyses (including β-lactams) demonstrated consistent intergroup efficacy. A further limitation is the exploratory nature of the subgroup analyses. Multiple comparisons were performed without statistical correction, which increases the risk of false-positive findings. Therefore, these results are considered hypothesis-generating and require confirmation in prospective studies. Finally, all participants were Chinese, which could limit generalizability due to possible differences in drug metabolism and pharmacokinetics. The results will have to be confirmed in other populations.

## Conclusions

This study demonstrated that levornidazole disodium phosphate treatment in Chinese patients with IAI was non-inferior to ornidazole in clinical cure rate, with levornidazole disodium phosphate exhibiting a more favorable safety profile than ornidazole.

## Electronic supplementary material

Below is the link to the electronic supplementary material.

Supplementary material 1

Supplementary material 2

## Data Availability

All data generated or analysed during this study are included in this published article and its supplementary information files.

## Abbreviation

ADRs: Adverse drug reactions
EOT: End of therapy
FAS: Full analysis set
IAIs: Intra-abdominal infections
ITT: Intention to treat
MI: Multiple imputation
NMPA: National Medical Products Administration
PPS: Per-protocol set
SS: Safety set
SAEs: Serious adverse events
TOC: Test of cure
TEAEs: Treatment-emergent adverse events
