# Supplementary material for: Efficacy and safety of levornidazole disodium phosphate injection in patients with intra-abdominal infections caused by anaerobic bacteria: a multicenter, randomized, single-blind, phase IV, non-inferiority trial (ANAEROGUARD study)
Source: BMC Infect Dis. 2026 Feb 28;26:719. doi: 10.1186/s12879-026-12789-7 (PMC13059180; doi:10.1186/s12879-026-12789-7)
Supplement: Supplementary file 2 — Supplementary material 2 [file 12879_2026_12789_MOESM2_ESM.docx]

**Supplementary materials**

**Inclusion criteria**

1. Adults aged 18 to 75 years (inclusive), irrespective of sex.
2. Patients scheduled to undergo minimally invasive surgery (laparoscopic or robot-assisted), open surgery, or percutaneous drainage for intra-abdominal infections deemed by the investigator to be caused by anaerobic bacteria.
3. Patients had to meet at least one of the following criteria: 1) at least two of the four preoperative indicators within 24 hours: i) abdominal pain or abdominal tenderness with fever (axillary temperature ≥37.5°C), ii) white blood cell count ≥10×10⁹/L, iii) elevated C-reactive protein above the upper limit of normal, or iv) elevated procalcitonin above the upper limit of normal, 2) radiological evidence of IAI confirmed by computed tomography or ultrasound.
4. Initiation or planned initiation of the investigational drug within 24 hours postoperatively.
5. Provision of written informed consent by the participant on a voluntary basis, with the consent process conducted in accordance with Good Clinical Practice (GCP) guidelines.

**Exclusion criteria**

A subject was excluded from enrollment if any of the following criteria were met:

1. Known or suspected hypersensitivity to nitroimidazole compounds.
2. Participation in other clinical trials and administration of investigational products within 3 months prior to screening.
3. Concomitant use of medications, or underlying conditions, that could interfere with the evaluation of the study drug’s safety or efficacy; or a high risk of serious drug–drug interactions (e.g., coadministration with warfarin or agents contraindicated with levoornidazole, such as furbenicillin sodium, nafcillin sodium, omeprazole, rabeprazole, potassium sodium dehydroandrographolide succinate for injection, or azlocillin sodium).
4. Considered by the investigator to have poor compliance or unlikely to complete the anticipated treatment course and follow-up.
5. Abnormal liver function (ALT or AST >1.5 times the ULN; in cases of acute biliary tract infection or hepatic abscess, the threshold was relaxed to >3× ULN), or renal impairment (creatinine clearance ≤60 mL/min/1.73 m², calculated by the Cockcroft-Gault formula).
6. Severe primary diseases involving major organs or systems (e.g., heart failure, leukemia, or uncontrolled diabetes mellitus) or malignancy.
7. Pregnant or lactating women, or women of childbearing potential with a positive pregnancy test prior to study entry.
8. Women of childbearing potential and male participants who were unable or unwilling to use effective contraception from the time of informed consent to 3 months after the last dose.
9. Concomitant infections at non-abdominal sites (chronic infections not expected to interfere with the evaluation of abdominal infection, such as chronic rhinitis or pharyngitis, were allowed).
10. Multi-organ failure.
11. Considered by the investigator to require treatment with broad-spectrum antibiotics with anti-anaerobic activity other than first-, second-, or third-generation cephalosporins, aztreonam, quinolones, or aminoglycosides.
12. Received antibiotic therapy within 48 hours prior to randomization (excluding a single dose).
13. Positive test results for hepatitis B surface antigen, HIV antibody, syphilis antibody, or hepatitis C antibody.
14. History of brain or spinal cord disorders, epilepsy, systemic sclerosis, hematopoietic dysfunction, or chronic alcoholism.
15. Any other condition deemed by the investigator to render the patient unsuitable for participation in this trial.

**Table S1. Bacteriological results and susceptibility results of antibiotics covering aerobic bacteria in the SS**

|  | **Visit** | **Results** | **Levornidazole group** (N=345) | **Control group** (N=345) |
| --- | --- | --- | --- | --- |
| Aerobic culture results | Baseline |  |  |  |
|  |  | n (Missing) | 322 (23) | 334 (11) |
|  |  | Negative, n (%) | 216 (67.08) | 222 (66.47) |
|  |  | Positive, n (%) | 106 (32.92) | 112 (33.53) |
|  | Postoperative day 1 |  |  |  |
|  |  | n (Missing) | 36 (309) | 31 (314) |
|  |  | Negative, n (%) | 26 (72.22) | 19 (61.29) |
|  |  | Positive, n (%) | 10 (27.78) | 12 (38.71) |
|  | Postoperative day 3 |  |  |  |
|  |  | n (Missing) | 38 (307) | 33 (312) |
|  |  | Negative, n (%) | 25 (65.79) | 18 (54.55) |
|  |  | Positive, n (%) | 13 (34.21) | 15 (45.45) |
|  | ETO |  |  |  |
|  |  | n (Missing) | 7 (338) | 10 (335) |
|  |  | Negative, n (%) | 5 (71.43) | 4 (40.00) |
|  |  | Positive, n (%) | 2 (28.57) | 6 (60.00) |
|  | TOC |  |  |  |
|  |  | n (Missing) | 3 (342) | 3 (342) |
|  |  | Negative, n (%) | 0 (0.00) | 1 (33.33) |
|  |  | Positive, n (%) | 3 (100.00) | 2 (66.67) |
| Anaerobic culture results | Baseline |  |  |  |
|  |  | n (Missing) | 309 (36) | 328 (17) |
|  |  | Negative, n (%) | 189 (61.17) | 217 (66.16) |
|  |  | Positive, n (%) | 120 (38.83) | 111 (33.84) |
|  | Postoperative day 1 |  |  |  |
|  |  | n (Missing) | 36 (309) | 30 (315) |
|  |  | Negative, n (%) | 28 (77.78) | 21 (70.00) |
|  |  | Positive, n (%) | 8 (22.22) | 9 (30.00) |
|  | Postoperative day 3 |  |  |  |
|  |  | n (Missing) | 36 (309) | 31 (314) |
|  |  | Negative, n (%) | 25 (69.44) | 21 (67.74) |
|  |  | Positive, n (%) | 11 (30.56) | 10 (32.26) |
|  | ETO |  |  |  |
|  |  | n (Missing) | 6 (339) | 9 (336) |
|  |  | Negative, n (%) | 5 (83.33) | 3 (33.33) |
|  |  | Positive, n (%) | 1 (16.67) | 6 (66.67) |
|  | TOC |  |  |  |
|  |  | n (Missing) | 3 (342) | 3 (342) |
|  |  | Negative, n (%) | 1 (33.33) | 1 (33.33) |
|  |  | Positive, n (%) | 2 (66.67) | 2 (66.67) |
| Susceptibility testing results | Baseline |  |  |  |
|  |  | n (Missing) | 113 (232) | 114 (231) |
|  |  | Negative, n (%) | 110 (97.35) | 109 (95.61) |
|  |  | Positive, n (%) | 3 (2.65) | 5 (4.39) |
|  | Postoperative day 1 |  |  |  |
|  |  | n (Missing) | 10 (335) | 11 (334) |
|  |  | Negative, n (%) | 10 (100.00) | 11 (100.00) |
|  |  | Positive, n (%) | 0 (0.00) | 0 (0.00) |
|  | Postoperative day 3 |  |  |  |
|  |  | n (Missing) | 13 (332) | 13 (332) |
|  |  | Negative, n (%) | 11 (84.62) | 13 (100.00) |
|  |  | Positive, n (%) | 2 (15.38) | 0 (0.00) |
|  | ETO |  |  |  |
|  |  | n (Missing) | 2 (343) | 6 (339) |
|  |  | Negative, n (%) | 2 (100.00) | 6 (100.00) |
|  |  | Positive, n (%) | 0 (0.00) | 0 (0.00) |
|  | TOC |  |  |  |
|  |  | n (Missing) | 3 (342) | 2 (343) |
|  |  | Negative, n (%) | 3 (100.00) | 2 (100.00) |
|  |  | Positive, n (%) | 0 (0.00) | 0 (0.00) |

EOT: end of therapy; TOC: test of cure.

**Table S2. Endpoints in the PPS**

| **Variables** | **Visit** | **Levornidazole group** | | **Control group** | | **Rate difference (95% CI)** |
| --- | --- | --- | --- | --- | --- | --- |
|  |  | **n (missing)** | **n (%)** | **n (missing)** | **n (%)** |  |
| Clinical cure rate | TOC | 314 (0) | 307 (97.77) | 317 (0) | 308 (97.16) | 0.61 (-2.05, 3.33) |
|  | EOT | 313 (1) | 305 (97.44) | 316 (1) | 307 (97.15) | 0.29 (-2.46, 3.07) |
| Bacteriological eradication rate | TOC | 107 (207) | 105 (98.13) | 102 (215) | 97 (95.10) | 3.03 (-2.43, 9.25) |
|  | EOT | 113 (201) | 108 (95.58) | 104 (213) | 100 (96.15) | -0.58 (-6.57, 5.59) |
| Overall success rate | TOC | 107 (207) | 105 (98.13) | 102 (215) | 97 (95.10) | 3.03 (-2.43, 9.25) |
|  | EOT | 113 (201) | 108 (95.58) | 104 (213) | 100 (96.15) | -0.58 (-6.57, 5.59) |

**Table S3. Sensitivity analysis of primary endpoint in the FAS**

| **Variables** | **Levornidazole group** | | **Control group** | | **Rate difference (95% CI)** |
| --- | --- | --- | --- | --- | --- |
|  | **n (missing)** | **n (%)** | **n (missing)** | **n (%)** |  |
| Sensitivity analysis of clinical cure rate | 334 (5) | 310 (92.81) | 336 (2) | 313 (93.15) | -0.34 (-4.32, 3.62) |

**Table S4. Bacteriological efficacy in the FAS**

| **Variables** | **Levornidazole group** (N=339) | **Control group** (N=338) |
| --- | --- | --- |
|  |  |  |
| N (Missing) | 117 (222) | 106 (232) |
| Eradication, n (%) | 0 (0.00) | 4 (3.77) |
| Presumed eradication, n (%) | 112 (95.73) | 98 (92.45) |
| Non-eradication, n (%) | 1 (0.85) | 1 (0.94) |
| Presumed non-eradication, n (%) | 4 (3.42) | 3 (2.83) |
| Overall eradication, n (%) | 112 (95.73) | 102 (96.23) |
| Rate difference (levornidazole group vs. control group) (95% CI) | -0.50 (-6.31, 5.54) |  |

**Table S5. Subgroup analysis (FAS)**

| **Variable** | **Levornidazole group** | | **Control group** | | **Rate difference between groups** | **95%CI** |
| --- | --- | --- | --- | --- | --- | --- |
|  | **n (missing)** | **n (%)** | **n (missing)** | **n (%)** |  |  |
| Age, years |  |  |  |  |  |  |
| <65 | 301 (5) | 280 (92.48) | 305 (2) | 287 (93.97) | -1.49 | (-5.73, 2.65) |
| >65 | 33 (0) | 30 (90.91) | 31 (0) | 26 (83.87) | 7.04 | (-10.03, 24.58) |
| Etiology |  |  |  |  |  |  |
| Appendicitis | 270 (5) | 251 (92.36) | 279 (2) | 259 (92.70) | -0.34 | (-5.00, 4.21) |
| Gastrointestinal perforation | 14 (0) | 12 (85.71) | 9 (0) | 7 (77.78) | 7.94 | (-22.25, 42.04) |
| Biliary tract infection or liver abscess | 50 (0) | 47 (94.00) | 48 (0) | 47 (97.92) | -3.92 | (-14.28, 5.74) |
| Surgical method |  |  |  |  |  |  |
| Minimally invasive surgery | 325 (5) | 303 (92.73) | 326 (2) | 306 (93.75) | -1.02 | (-5.09, 2.97) |
| Open surgery | 4 (0) | 3 (75.00) | 6 (0) | 4 (66.67) | 8.33 | (-42.45, 50.31) |
| Percutaneous drainage | 5 (0) | 4 (80.00) | 4 (0) | 3 (75.00) | 5.00 | (-42.11, 52.83) |
| Concomitant aerobic antibiotic class |  |  |  |  |  |  |
| Quinolones | 32 (0) | 30 (93.75) | 23 (0) | 20 (86.96) | 6.79 | (-9.50, 26.41) |
| Aminoglycosides | 1 (0) | 1 (100.00) | 0 | 0 (0) | / | / |
| β-lactams or others | 206 (0) | 197 (95.63) | 216 (0) | 211 (97.69) | -2.05 | (-6.01, 1.57) |
| Age > 60 years | 52 (0) | 48 (92.31) | 50 (0) | 43 (86.00) | 6.31 | (-6.32, 19.35) |
| Suppurative appendicitis | 233 (3) | 217 (92.80) | 229 (2) | 214 (93.29) | -0.49 | (-5.34, 4.36) |
| Gangrenous perforated appendicitis | 27 (2) | 24 (86.21) | 30 (0) | 27 (90.00) | -3.79 | (-23.07, 14.05) |
| Appendix periappendiceal abscess | 9 (0) | 9 (100.00) | 17 (0) | 15 (88.24) | 11.76 | (-19.33, 34.34) |
| Age ≥65 years with etiology of appendicitis | 26 (0) | 25 (96.15) | 19 (0) | 14 (73.68) | 22.47 | (1.57, 45.17) |
| Age >45 years, etiology of appendicitis or gastrointestinal perforation, no use of β-lactam or other combined aerobic antibiotics | 26 (1) | 23 (87.78) | 25 (0) | 16 (64.00) | 23.78 | (3.78, 68.25) |

CI: confidence interval; EOT: end of therapy; TOC: test of cure.

**Table S6. Post hoc subgroup analysis**

| **Subgroup** | **Levornidazole group** | | **Control group** | | **Risk difference (95% CI)** |
| --- | --- | --- | --- | --- | --- |
|  | **n (missing)** | **n (%)** | **n (missing)** | **n (%)** |  |
| Age >60 years | 52 (0) | 48 (92.31) | 50 (0) | 43 (86.00) | 6.31 (-6.32, 19.35) |
| Suppurative appendicitis | 233 (3) | 217 (92.80) | 229 (2) | 214 (93.29) | -0.49 (-5.34, 4.36) |
| Gangrenous or perforated appendicitis | 27 (2) | 24 (86.21) | 30 (0) | 27 (90.00) | -3.79 (-23.07, 14.05) |
| Periappendiceal abscess | 9 (0) | 9 (100.00) | 17 (0) | 15 (88.24) | 11.76 (-19.33, 34.34) |
| Age ≥65 years with appendicitis | 26 (0) | 25 (96.15) | 19 (0) | 14 (73.68) | 22.47 (1.57, 45.17) |
| Age >45 years, with appendicitis or gastrointestinal perforation, without β-lactam or other use | 26 (1) | 23 (87.78) | 25 (0) | 16 (64.00) | 23.78 (3.78, 68.25) |

**Figure S1. Forest plot of post hoc subgroup analysis**

**
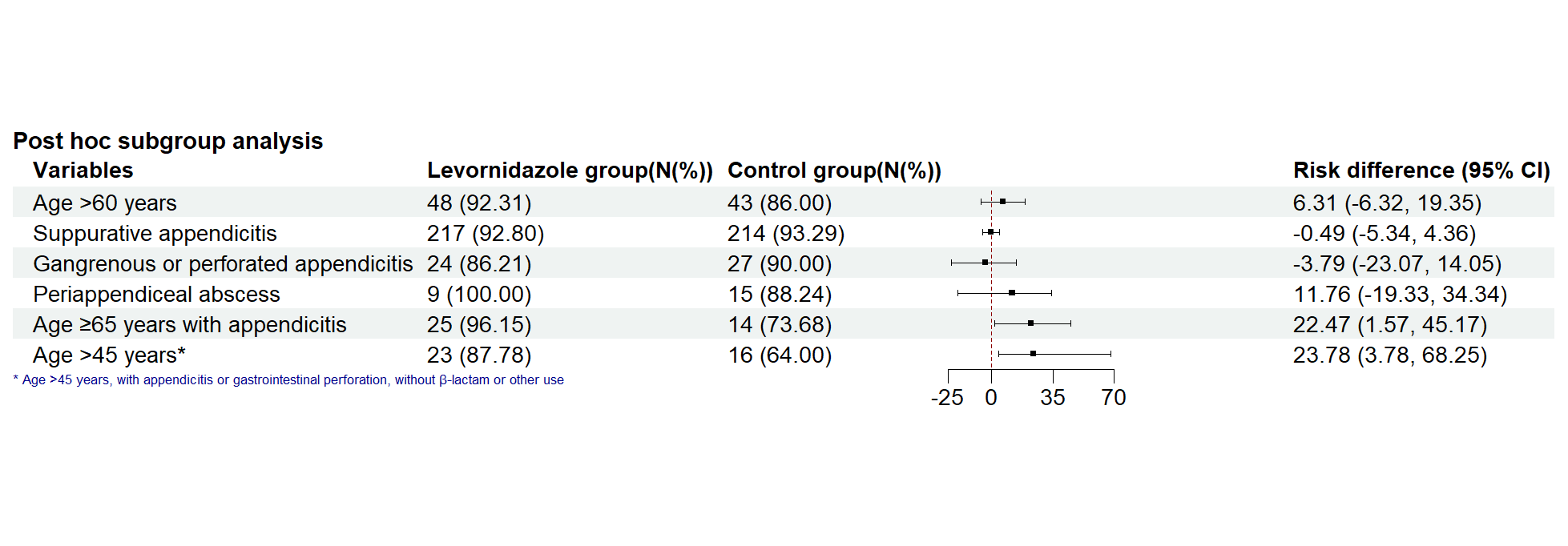
**
